# CircDENND2A Promotes Non-small Cell Lung Cancer Progression via Regulating MiR-34a/CCNE1 Signaling

**DOI:** 10.3389/fgene.2020.00987

**Published:** 2020-09-01

**Authors:** Yinbin Zhang, Changyou Shan, Yinxi Chen, Shiyu Sun, Di Liu, Xin Zhang, Shuqun Zhang

**Affiliations:** Department of Oncology, The Second Affiliated Hospital of Xi’an Jiaotong University, Xi’an, China

**Keywords:** circDENND2A, non-small cell lung cancer, biomarker, miR-34a, CCNE1

## Abstract

The mechanism regulating non-small cell lung cancers (NSCLCs) is unclear. In this study, we aimed to determine the roles of DENN domain containing 2A (circDENND2A) in the progression of NSCLC. Circular RNAs (circRNAs) are composited by “head to tail” splicing of coding or non-coding RNAs (ncRNAs), whose crucial roles in human cancers had been revealed. CircDENND2A, a new circRNA, was revealed to induce cell proliferation and migration. Our data indicated that circDENND2A was a probable oncogene in human cancers. However, the roles of circDENND2A in NSCLC remained unknown. Here, we demonstrated that circDENND2A was down-regulated in NSCLC samples. Loss-of-function assays showed circDENND2A knockdown suppressed cell growth via inducing cell cycle arrest and apoptosis and inhibited cell migration and invasion. Bioinformatics analysis and competing endogenous RNA (ceRNA) network analysis revealed that circDENND2A was involved in regulating cell cycle and tumor protein p53 (TP53) signaling via miR-34a/CCNE1 (cyclin E1). Further validation showed that circDENND2A could directly bind to miR-34a, promoting CCNE1 expression in NSCLC. In addition, rescue assays demonstrated that restoration of CCNE1 significantly impaired the suppressive effects of circDENND2A silencing in terms of NSCLC growth, migration, and invasion. We thought this study indicated that circDENND2A/miR-34a/CCNE1 may be a potential therapeutic target for NSCLC.

## Introduction

The proportion of non-small cell lung cancers (NSCLCs) was about 85% among lung cancer (Gu et al., 2018; Luo et al., 2020). Although great progress has been made in the related researches on lung cancer, 5-year overall survival (OS) time in patients with NSCLC was not more than 15% because of distant metastasis in numerous NSCLC patients (Scheff and Schneider, 2013; Gu et al., 2016). Multiple “drivers” were identified to be related to the tumor genesis and metastasis of NSCLC, including protein, microRNAs (miRNAs), and long non-coding RNAs (lncRNAs) (Bousema et al., 2020; Chen Z. et al., 2020; Lee et al., 2020; Marin et al., 2020; No Authors, 2020). For example, GIAT4RA was found to be a carcinoma inhibitor in NSCLC via offsetting Uchl3-induced de-ubiquitination of LSH (Yang et al., 2019). A recent study demonstrated that LINC01234 promoted cell metastasis of NSCLC via causing VAV3 activation but BTG2 inhibition (Chen Z. et al., 2020). However, the molecular mechanisms regulating NSCLC metastasis are not well-understood.

Circular RNAs (circRNAs) have the characteristics of covalently closed continuous-loop shape (Wei et al., 2019; Kong et al., 2020). Multiple circRNAs were identified in human cells by virtue of high-throughput sequencing toolset. Increasing evidences demonstrated that circRNAs were involved in regulating lung cancer cell viability, apoptosis, autophagy, and invasion (Di et al., 2019). For instance, circPTPRA induced repression of epithelial–mesenchymal transition and NSCLC cell metastasis by sponging miR-96-5p (Wei et al., 2019). CircHIPK3 regulated autophagy by MIR124-3p-STAT3-PRKAA/AMPKα signaling in lung cancer with mutated STK11 (Chen X. et al., 2020) and retarded NSCLC apoptosis by sponging miR-149 (Lu et al., 2020). Down-regulation of circRNA ciRS-7 resulted in NSCLC apoptosis (Su et al., 2018). To explore unknown molecular functions and roles of circRNAs in NSCLC is therefore extremely important, suggesting it would be helpful for us to identify novel biomarkers for NSCLC.

DENND2A is a member of guanine nucleotide exchange factor (GEF), which may activate RAB9A and RAB9B. DENND2A may play a role in late endosomes back to trans-Golgi network (TGN) transport. CircDENND2A, a new circRNA, was revealed to induce cell proliferation and migration but declined apoptosis in H9C2 cells (Shao et al., 2020). Meanwhile, circDENND2A enhanced migration and invasion of glioma cells (Su et al., 2019). Our data indicated that circDENND2A was a probable oncogene in human cancers. However, the roles of circDENND2A in NSCLC remained to be unknown. Here, we identified the circDENND2A expression level in NSCLC specimens and revealed the potential functions of this circRNA in NSCLC using loss-of function assays. Up to date, we are the first to reveal that circDENND2A was a newly produced indicator for prognosis and a promising target in NSCLC therapy.

## Materials and Methods

### Patients and Samples

In total, 20 pairs of NSCLC tissues and adjacent normal ones in treated NSCLC patients (from Jan 2013 to Jan 2016) were collected from our hospital. Our experiments were conducted after the approval of the Board and Ethics Committee of our hospital. All the subjects unanimously consented with written informed documents. All the harvested samples were kept in liquid nitrogen for the following use.

### Cell Culture

NSCLC cells including A549 and H1299 were ordered from the American Type Culture Collection (ATCC) (Maryland, United States) and kept in Roswell Park Memorial Institute (RPMI) 1640 medium (GINSCLCO, Thermo Fisher Scientific, Inc., Waltham, MA, United States) supplied by 10% fetal bovine serum (FBS) and 1% penicillin/streptomycin (HyClone; GE Healthcare Life Sciences, Logan, UT, United States) at 37°C incubator with 5% CO_2_.

### Extraction and Quantitation of RNA

TRIzol (Thermo Fisher Scientific, United States) was applied to extract whole RNA from tissues and cells as the manufacturer described. Nanodrop spectrophotometer (Thermo Fisher Scientific, United States) was performed to quantify RNA. Reverse Transcription Kit (TaKaRa, Japan) was used to perform reverse transcription for circRNA and mRNA per the manufacturer’s instruction. Quantitative real-time PCR (qRT-PCR) was run on Mx3005P QPCR Systems (Agilent Technologies, Inc., United States) as the manual described. GAPDH and small nuclear U6B were separately considered as internal reference for circRNAs and miRNAs. Primers used in this part were derived from Biosune (Shanghai, China). 2^–ΔΔCt^ method was applied to calculate relative gene expression. The primers are as follows: circDENND2A forward, 5′-TGAACAGAAGACTGTGGACCG-3′ and reverse, 5′-CAGTCTCTAGGAATGGAATGGAGG-3′; DENND2A forward, 5′-AACTGAAGGCCATTCCCCAG-3′ and reverse, 5′-TCTTCGGCAGTAACCGAACC-3′; β-actin forward, 5′-ATCATTGCTCCTCCTGAGCG-3′ and reverse, 5′-ACTCCTGCTTGCTGATCCAC-3′; and DENND2A for- ward, 5′-CGGCTCGCTCCAGGAA-3′ and reverse, 5′-TCATCT GGATCCTGCAAAAAAA-3′.

### Cell Viability Assay

A total of 3 × 10^3^ of siRNA-transfected cells in each well were inoculated in 96-well plates overnight. At the following day, Cell Counting Kit-8 (CCK-8) kit (Promega, United States) was applied to detect cell viability as indicated by the manufacturer. The optical density (OD) value of 492 nm was detected with the use of Microplate Reader (Multiskan Sky, Thermo Fisher Scientific).

### Competing Endogenous RNA Analysis and Target Prediction

We predicted the circRNA/miRNA interaction using the CircNet database^1^, and we constructed a circRNA–miRNA–gene regulatory networks using the Cytoscape software. The potential miRNA binding sites on circRNAs were predicted through RNA22 v2 and RNAhybrid.

### Migration and Invasion Assay

Cell migration and invasion were detected in BD 24-well Transwell chamber (Costar, Massachusetts, United States) in the presence or absence of pre-coated Matrigel as the manual declared. A total of 6 × 10^4^ cells in 500 μl of medium without serum were put to insert, and the corresponding volume of the medium with 10% FBS as chemoattractant was set in a lower chamber. At the following day, cells with invasion that occurred at the lower surface of the insert were fixed and dyed with 1% crystal violet, followed by counting and capturing.

### Flow Cytometry Assay

Flow cytometry assay (FACSCalibur, BD Biosciences) was applied to detect cell apoptosis and cell cycle. Annexin V-FITC Apoptosis Kit (BD Biosciences) was used to measure cell apoptosis as the manual suggested. Propidium iodide (PI) along with Ribonuclease A (Sigma) was added into cells to measure cell cycle progression as referred to in the protocol of the manual.

### Luciferase Reporter Assay

PGL3-Basic luciferase construct (GenePharma, Shanghai, China) containing circDENND2A-WT, circDENND2A-mutant, CCNE1-WT, and CCNE1-mutant was used as a reporter expression cassette to execute luciferase reporter assay. The above-mentioned reporter expression cassette and microRNAs were co-transfected into cells by Lipofectamine 2000 (Life Technologies). Dual-luciferase reporter assay system (Promega, Madison, WI, United States) was consequently performed to determine luciferase activity after relative to internal control renilla after 48-h transfection.

### Western Blot Analysis

Harvested cells were lysed by radioimmunoprecipitation assay (RIPA) buffer. Protein separation was executed by sodium dodecyl sulfate–polyacrylamide gel electrophoresis (SDS-PAGE) analysis, followed by transferring to polyvinylidene difluoride (PVDF) membranes and incubating with respective primary antibodies overnight, such as 1:1,000 diluted anti-GAPDH antibody (Sigma) and anti-CCNE1 antibody (ab138222, Abcam). Enhanced chemical luminescence solution (32109; Thermo Fisher Scientific) was used for detecting band intensity.

### Statistical Analysis

GraphPad Prism 7.0 (GraphPad, La Jolla, CA, United States) was conducted to perform statistical analysis. Our data were represented as mean ± SD (standard deviation). The difference of the two compared groups was counted by Student *t*-test. The progression-free survival (PFS) and OS between two groups was analyzed by Kaplan–Meier method and the log-rank test. Significant difference means *P*-value is not more than 0.05.

## Results

### CircDENND2A Level Was Largely Up-Regulated While DENND2A Level Was Decreased in Non-small Cell Lung Cancer Tissues

**FIGURE 1:**
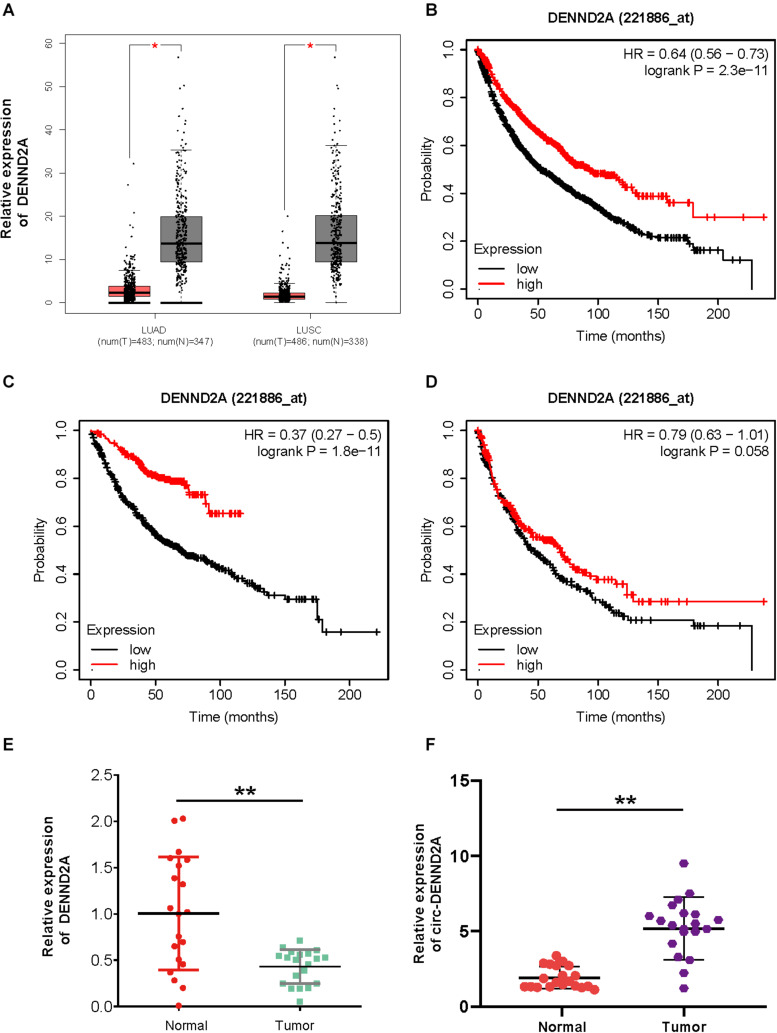
CircDENND2A is up-regulated in NSCLC. **(A)** GEPIA database indicated DENND2A was up-regulated in NSCLC samples compared with normal tissues. **(B–D)** Kaplan–Meier analysis demonstrated that higher expression levels of DENND2A correlated to longer survival time in patients with NSCLC, LUAD, and LUSC by using Kaplan–Meier plotter database. **(E,F)** The high expression levels of DENND2A **(E)** and circDENND2A **(F)** in 20 paired NSCLC tissues by qRT-PCR (**P < 0.01). NSCLC, non-small cell lung cancer; GEPIA, Gene Expression Profiling Interactive Analysis; LUAD, lung adenocarcinoma; LUSC, lung squamous cell carcinoma.

We firstly detected the expression pattern of DENND2A in NSCLC, which was the corresponding liner RNA of circDENND2A. As presented in Figure 1, we found that DENND2A was significantly suppressed in lung adenocarcinoma (LUAD) and lung squamous cell carcinoma (LUSC) samples compared with normal tissues (Figure 1A). Kaplan–Meier analysis demonstrated that higher expression levels of DENND2A were correlated to longer survival time in patients with NSCLC, LUAD, and LUSC by using the Kaplan–Meier plotter database (Figures 1B–D). These findings suggested that DENND2A may serve as a tumor suppressor.

To further validate the above findings, we performed qRT-PCR analysis to detect circDENND2A and DENND2A expression patterns in NSCLC tissues and adjacent normal ones of 20 NSCLC patients. Figure 1C demonstrates that DENND2A expression was hugely suppressed in NSCLC tissues when compared with that in adjacent normal ones (Figure 1C). However, circDENND2A was up-regulated in NSCLC samples (Figure 1D). These results showed circDENND2A may have an opposite role in NSCLC as compared with DENND2A.

### Reduced CircDENND2A Retarded Non-small Cell Lung Cancer Cell Viability, Migration, and Invasion

**FIGURE 2:**
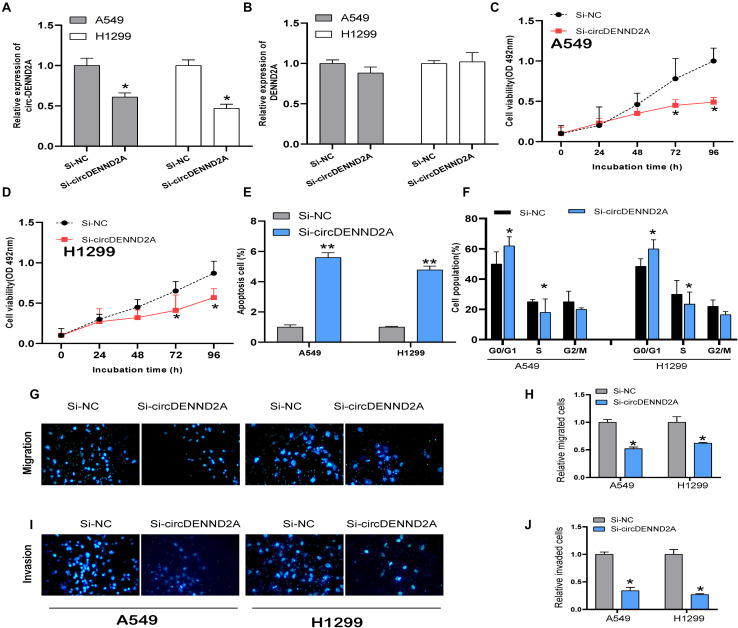
CircDENND2A promoted tumorigenesis in NSCLC cells. **(A,B)** Expression of circDENND2A and DENND2A was confirmed by qRT-PCR in A549 and H1299 cells transfected with Si-NC or Si-circDENND2A. **(C,D)** Knockdown of circDENND2A significantly inhibited cell proliferation of A549 **(C)** and H1299 **(D)** cells by CCK-8 assay. **(E)** Cell apoptosis was determined by flow cytometry in A549 and H1299 cells transfected with Si-NC or Si-circDENND2A. **(F)** Cell cycle was determined by flow cytometry in A549 and H1299 cells transfected with Si-NC or Si-circDENND2A. **(G–J)** Transwell assay was used to detect the migration and invasion of A549 **(G,H)** and H1299 **(I,J)** cells transfected with Si-NC or Si-circDENND2A. Our data were represented as mean ± SD (standard deviation) (*P < 0.05, **P < 0.01). NSCLC, non-small cell lung cancer; CCK-8, Cell Counting Kit-8.

A549 and H1299 cells were selected to conduct loss-of-function assay and further explored whether circDENND2A exerted effects on NSCLC cell biological function. For that purpose, we firstly performed qRT-PCR analysis to verify that circDENND2A expression was indeed ablated after transfection of siRNA (Figure 2A). However, the expression of DENND2A was not affected after transfection of siRNA (Figure 2B). Cell Counting Kit-8 (CCK-8) assay was then executed and demonstrated that reduced circDENND2A immensely repressed capacity of A549 (Figure 2C) and H1299 cell viability (Figure 2D). Flow cytometry assessment was carried out to validate the effects on NSCLC cell apoptosis (Figure 2E) and cycle (Figure 2F) induced by circDENND2A. Our data indicated that down-regulation of circDENND2A largely induced A549 and H1299 cell apoptosis (Figure 2E) and restrained cell cycle at G0/G1 stage (Figure 2F). Transwell assay demonstrated that decreased circDENND2A resulted in greatly attenuated ability of NSCLC cell migration (Figures 2G,H) and invasion (Figures 2I,J).

### Bioinformatics Analysis of CircDENND2A

**FIGURE 3:**
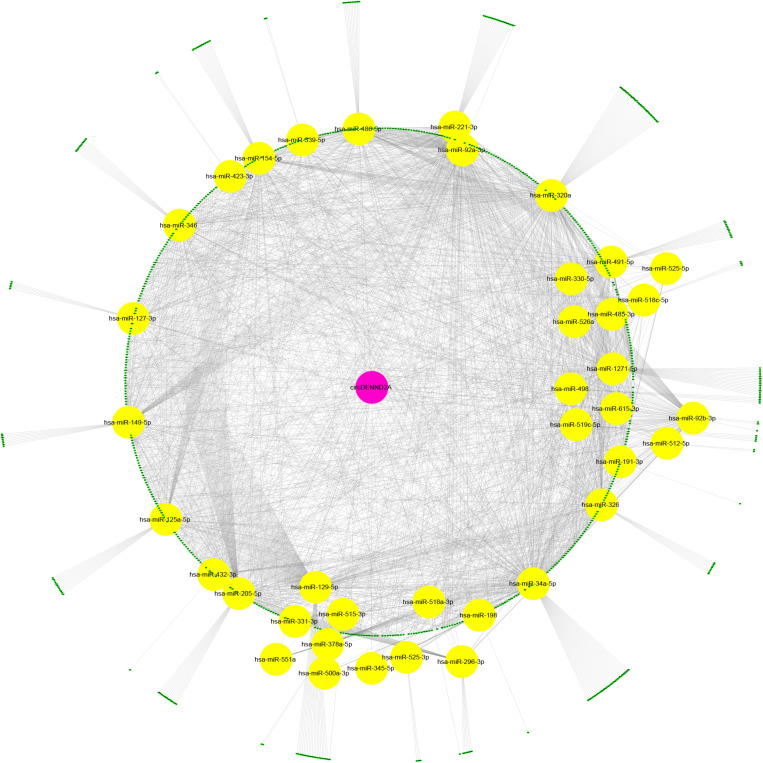
Construction of circDENND2A associated ceRNA network. CircDENND2A associated ceRNA networks included 39 miRNAs and 867 differently expressed mRNAs. Purple node, circDENND2A; yellow nodes, miRNAs; blue nodes, mRNAs. ceRNA, competing endogenous RNA; miRNA, microRNA.

We performed bioinformatics analysis to uncover the mechanisms of circDENND2A in NSCLC. Previous studies revealed that circRNAs-mediated modulation of miRNA level via sponging miRNA. To uncover whether circDENND2A sponged miRNA to regulate NSCLC cell progress, candidate miRNAs related to circDENND2A were forecasted by public bioinformatics tool Starbase^2^ and CircInteractome^3^, as previous study described (Gu et al., 2020). A total of 9,865 potential targets of circDENND2A were obtained. Then, we extracted different expressed genes in NSCLC using GEPIA datasets. Finally, we constructed circDENND2A associated competing endogenous RNA (ceRNA) networks, which included 39 miRNAs and 867 differently expressed mRNAs (Figure 3).

**FIGURE 4:**
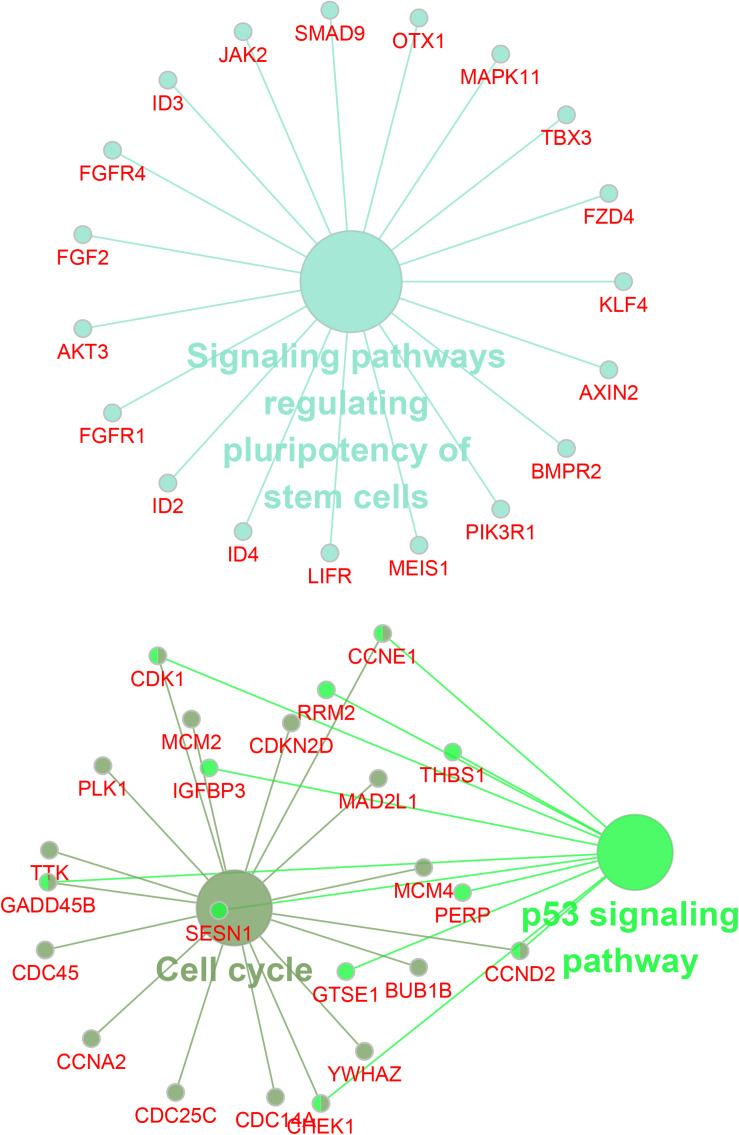
Bioinformatics analysis of circDENND2A associated ceRNA network. CircDENND2A associated ceRNA networks included 39 miRNAs and 867 differently expressed mRNAs. Purple node, circDENND2A; yellow nodes, miRNAs; blue nodes, mRNAs. ceRNA, competing endogenous RNA; miRNA, microRNA.

Then, we employed bioinformatics analysis for this network. As presented in Figure 4, the results showed that circDENND2A was involved in signaling pathways regulating pluripotency of stem cells via OTX1, LIFR, SMAD9, JAK2, MAPK11, FZD4, KLF4, TBX3, AXIN2, BMPR2, ID4, ID2, ID3, FGFR1, AKT3, FGF2, FGFR4, PIK3R1, and MEIS1, and also related to cell cycle and TP53 signaling via CHEK1, RRM2, YWHAZ, GTSE1, CCNE1, MAD2L1, CDC14A, CDKN2D, PLK1, TTK, GADD45B, CDC45, CCNA2, CDK1, SESN1, CDC25C, MCM2, IGFBP3, BUB1B, MCM4, PERP, CCND2, and THBS1.

### CircDENND2A Sponged MiR-34a and Induced CCNE1 Expression

**FIGURE 5:**
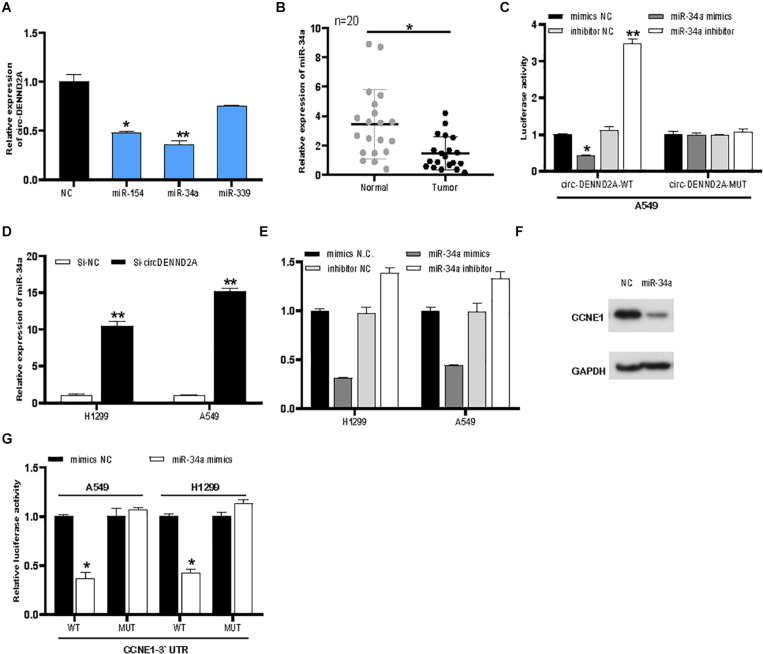
CircDENND2A directly interacted with miR-34a and promoted CCNE1 expression. **(A)** CircDENND2A expression was detected after overexpression of miR-154, miR-34a, and miR-339. **(B)** The expression of miR-34a in tumor tissues was significantly down-regulated. **(C)** The relative luciferase activities were detected in the A549 transfected with the miR-34a or miR-34a inhibitors, reporter vector WT-circDENND2A, or MUT-circDENND2A. **(D)** The expression of miR-34a was inhibited in A549 and H1299 cells transfected with Si-circDENND2A. **(E)** The expression of CCNE1 was determined by qRT-PCR in A549 and H1299 cells transfected with NC, miR-34a inhibitor, and miR-34a mimics. **(F)** Western blot assay showed that miR-34a mimics could suppress CCNE1 level in A549 cells. **(G)** The relative luciferase activities were detected in the A549 transfected with the miR-34a or miR-34a inhibitors, reporter vector WT-CCNE1, or MUT-CCNE1 (*P < 0.05, **P < 0.01).

The present study focused on exploring the effect of circDENND2A on CCNE1, which is a key cell cycle regulator. Three candidate miRNAs (miR-154, miR-339, and miR-34a) were predicted to target CCNE1 and circDENND2A, which acted as tumor inhibitors. qRT-PCR assay showed that overexpression of miR-154 and miR-34a suppressed circDENND2A levels (Figure 5A). Previous reports revealed that miR-34a was a tumor inhibitor and that its expression was weak in numerous cancers (Li et al., 2018; Haghi et al., 2019; Luo et al., 2019). Our data verified that miR-34a level was down-regulated in NSCLC tissues (Figure 5B). Subsequent dual-luciferase reporter assay was carried out to make forward to exploring whether circDENND2A possessed direct interaction with miR-34a. Our data illustrated that miR-34a mimics could greatly abolish the luciferase activity of the WT-circDENND2A instead of that of MUT-circDENND2A (Figure 5C). The opposite conclusion was demonstrated in miR-34a inhibitor-treated A549 and H1299 cells (Figure 5C). Simultaneously, reduced circDENND2A expression level notably enhanced miR-34a expression level (Figure 5D), revealing that miR-34a was modulated by circDENND2A and was downstream target of circDENND2A.

Our analysis suggested that miR-34a possessed binding site of CCNE1. Finally, miR-34a mimics could hinder CCNE1 level in NSCLC cells (Figure 5E). However, knockdown of miR-34a enhanced CCNE1 expression in A549 and H1299 (Figure 5E). Western blot assay showed that miR-34a mimics could suppress CCNE1 level in A549 cells (Figure 5F). We then conducted luciferase reporter assay to deeply unearth the interaction between miR-34a and CCNE1. Our data showed that miR-34a mimics decreased WT-CCNE1 3′-UTR activity instead of MUT-CCNE1 3′-UTR activity (Figure 5G). Our data revealed that circDENND2A mediated heightened CCNE1 expression in NSCLC cells through sponging miR-34a.

### Rescued CCNE1 Reversed Decreased CircDENND2A-Mediated Hindered Effects on Non-small Cell Lung Cancer Cell

**FIGURE 6:**
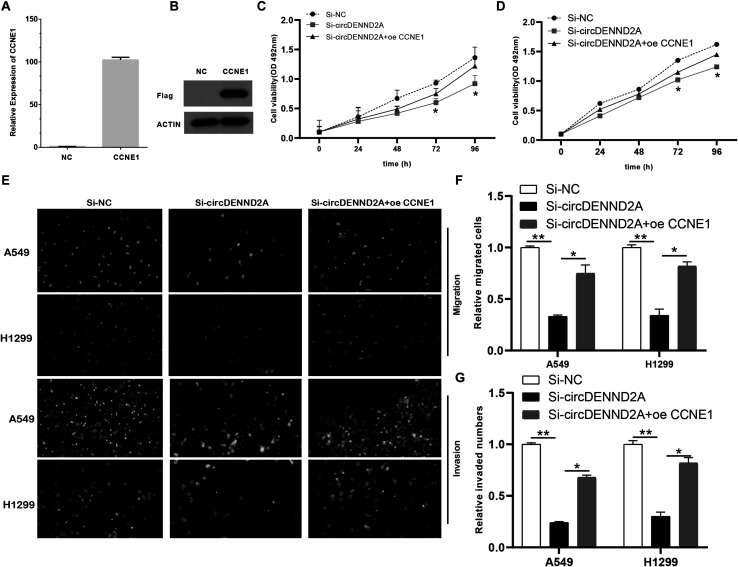
Restoration of CCNE1 impaired the inhibitory effects of circDENND2A silencing. **(A,B)** The mRNA **(A)** and protein **(B)** expression of CCNE1 was determined in A549 cells of circDENND2A knockdown. **(C,D)** CCK-8 assays demonstrated that CCNE1 overexpression could largely reverse the suppressive effects of circDENND2A silencing on proliferation of A549 and H1299 cells. **(E–G)** CCNE1 overexpression partly reversed the suppressive effects of circDENND2A silencing on migration and invasion of A549 and H1299 cells (*P < 0.05, **P < 0.01). CCK-8, Cell Counting Kit-8.

Then, we applied gain-of-function assays for CCNE1 in NSCLC cells. The results showed that both RNA and protein levels of CCNE1 were up-regulated in A549 cells after overexpressing CCNE1 overexpression plasmids (Figures 6A,B). To further validate if circDENND2A functioned via regulation of CCNE1 expression, we overexpressed CCNE1 in circDENND2A-knockdown NSCLC cells. As presented in Figures 6C,D, CCK-8 assay indicated that rescued CCNE1 level could evidently reverse the influence of down-regulated circDENND2A-induced restraint NSCLC cell growth (Figures 6C,D). Additionally, Transwell assay displayed that overexpression of CCNE1 could undermine inhibitory influence of decreased circDENND2A-mediated cell migration and invasion of NSCLC (Figures 6E–G). Collectively, our data demonstrated that circDENND2A actuated cell viability, migration, and invasion via increasing CCNE1 level through sponging miR-34a in NSCLC.

### CCNE1 Level Was Up-Regulated in Non-small Cell Lung Cancer Tissues

**FIGURE 7:**
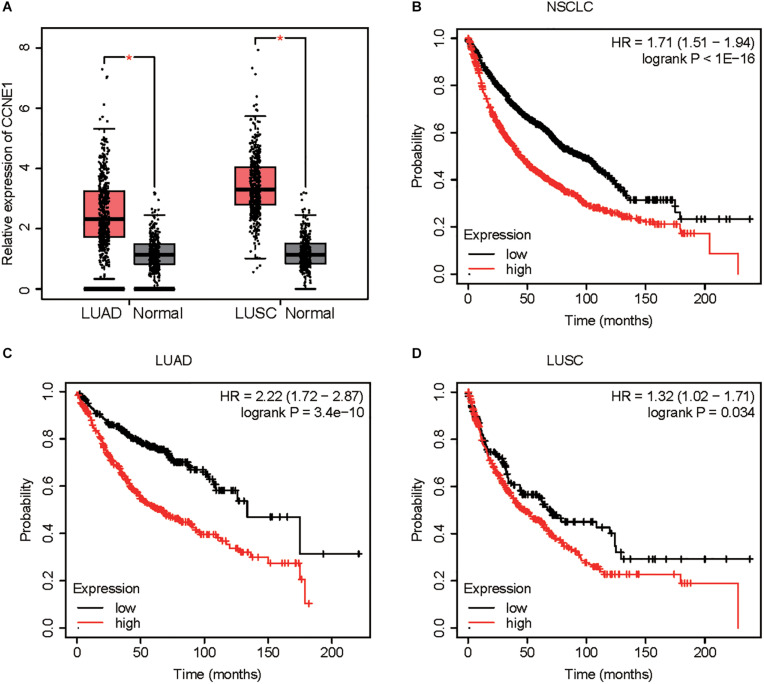
CCNE1 is up-regulated in NSCLC. **(A)** GEPIA database indicated CCNE1 was up-regulated in NSCLC samples when compared with normal tissues. **(B–D)** Kaplan–Meier analysis demonstrated that higher expression levels of CCNE1 correlated with longer survival time in patients with NSCLC, LUAD, and LUSC by using the Kaplan–Meier plotter database (*P < 0.05). NSCLC, non-small cell lung cancer; GEPIA, Gene Expression Profiling Interactive Analysis; LUAD, lung adenocarcinoma; LUSC, lung squamous cell carcinoma.

We detected the expression pattern of CCNE1 in NSCLC. As presented in Figure 1, we found that CCNE1 was significantly suppressed in LUAD and LUSC samples compared with normal tissues (Figure 7A). Kaplan–Meier analysis demonstrated that higher expression levels of CCNE1 were correlated to longer survival time in patients with NSCLC, LUAD, and LUSC using the Kaplan–Meier plotter database (Figures 7B–D).

## Discussion

CircRNAs are composited by “head to tail” splicing of coding or non-coding RNAs (ncRNAs) (Bonnici et al., 2014). Increasing evidences demonstrated that circRNAs may play different even opposite effects on the cancer progression compared with their parental genes. For example, current studies have shown that FOXO3a was a carcinoma inhibitor (Warr et al., 2013; Liu et al., 2015). However, Kong et al. (2019) identified that circFOXO3 impelled prostate cancer development via sponging miR-29a-3p, and Zhang et al. (2019) revealed that circFOXO3 caused glioblastoma progress by competitive action with endogenous RNA for NFAT5. CircDENND2A was a novel circRNA and promoted glioma progression (Su et al., 2019). However, the expression levels and molecular functions of this circRNA in NSCLC remained largely unclear. The present study revealed that circDENND2A was up-regulated; however, DENND2A was down-regulated in 15 paired NSCLC samples. DENND2A belonged to class of DEEND2 gene family and was reported to be a particular GEF, which activated RAB9A and RAB9B and participated in trafficking between the TGN and late endosomes (Yoshimura et al., 2010). Previous studies indicated that DENND2A was related to ischemic stroke and Parkinson’s disease (Lang et al., 2019). Nevertheless, the function of DENND2A in cancers remained elusive. In order to further validate these findings, we analyze DENND2A mRNAs levels between NSCLC samples and normal lung tissues by using public databases. We found that DENND2A was significantly reduced in both LUAD and LUSC. Higher expression levels of DENND2A were correlated to longer OS and PFS time in patients with NSCLC.

CircDENND2A had been demonstrated to be an oncogene in glioma and enhanced glioma cell migration and invasion (Su et al., 2019; Shao et al., 2020). In myocardial ischemia model, circDENND2A was involved in promoting cell viability and migration but declining apoptosis of H9c2 (Shao et al., 2020). Our study firstly showed circDENND2A served as an oncogene in NSCLC through CCK-8 assay and Transwell assay. Ablated circDENND2A largely reduced biological performance of NSCLC cells. Emerging reports have manifested that circRNAs post-transcriptionally affected the expression of downstream targets through sponging miRNAs and acted as ceRNAs. For example, circPTPRA obstructed transition of epithelial–mesenchymal and metastasis via sponging miR-96-5p in NSCLC cells (Wei et al., 2019). CircRNA HIPK3 induced cell proliferation but repressed apoptosis in NSCLC through sponging miR-149 (Lu et al., 2020). CircRNA hsa_circ_0023404 induced viability, migration, and invasion in NSCLC via sponging miR-217 to enhance ZEB1 expression (Liu et al., 2019). In our study, bioinformatics analysis and dual-luciferase reporter experiments verified that circDENND2A directly targeted to miR-34a. Moreover, we found that decreasing circDENND2A largely raised miR-34a level. miR-34a was found to suppress cancer development in colon cancer (Sun et al., 2018), gastric cancer (Zhang et al., 2020), osteosarcoma, pancreatic ductal adenocarcinoma, breast cancer, and prostate cancer. In NSCLC, miR-34a was found to inhibit tumor genesis via targeting SIRT6 (Ruan et al., 2018), hindering cell viability, inducing apoptosis, and blocking cell-cycle progress through epidermal growth factor receptor (EGFR) (Yin et al., 2013) and to modulate ionizing radiation-induced senescence via targeting c-MYC and regulating gefitinib-acquired resistance via targeting Axl. These reports suggesting that miR-34a played a crucial role in NSCLC.

Then, bioinformatics analysis and luciferase reporter experiment displayed that miR-34a targeted to CCNE1 and performed negative regulation of its level. CCNE1 functioned as an elementary modulator in G1/S cell cycle (Wang et al., 2009). Aberrant increased CCNE1 indeed enhanced CDK2 conversely resulting in substrate phosphorylation of pRb, thus causing peculiar cell viability (Neganova et al., 2011). CCNE1 is found to be an oncogene in multiple cancer types, such as gastric cancer, colorectal cancer, prostate cancer, and NSCLC (Etemadmoghadam et al., 2010; Amininia et al., 2014; Pils et al., 2014; Kim et al., 2016; Noske et al., 2017). MiRNAs were considered as important regulators of CCNE1. MiR-15b (Yuan et al., 2019), miR-195 (Wang et al., 2019), and miR-34a (Han et al., 2015) were reported to regulate CCNE1 expression in multiple cancers. Here, our data revealed that miR-34a controlled CCNE1 expression level by targeting to CCNE1. Nevertheless, circDENND2A sponged miR-34a to induce CCNE1 expression in NSCLC cells. Moreover, rescue experiment indicated that circDENND2A played an oncogenic part in CCNE1-dependent way.

Several limitations should be noted in this study. First, the correlations between circDENND2A expression and survival time in NSCLC remained unclear. In the future study, we will collect more clinical samples to analyze the correlations among them. Second, the present study aimed to explore the potential functions of circDENND2A in NSCLC. However, the detailed mechanisms of DENND2A in NSCLC remained to be further investigated. The loss- or gain-of-function assays should be applied in the future study.

In summary, our study firstly demonstrated that circDENND2A is an oncogene in NSCLC. More importantly, our data illustrated that reduced circDENND2A protected cell viability and migration via targeting miR-34a/CCNE1 axis, revealing a newly generated target on circDENND2A in NSCLC therapy.

## Data Availability Statement

The raw data supporting the conclusions of this article will be made available by the authors, without undue reservation, to any qualified researcher.

## Author Contributions

YZ and SZ designed the project and were responsible for the overall content. CS performed bioinformatics analysis. YC, SS, DL, and XZ carried out all experiments. YZ and CS prepared the manuscript. SZ contributed to revise the manuscript. All authors contributed to the article and approved the submitted version.

## Conflict of Interest

The authors declare that the research was conducted in the absence of any commercial or financial relationships that could be construed as a potential conflict of interest.

## Footnotes

1 http://circnet.mbc.nctu.edu.tw/

2 http://starbase.sysu.edu.cn/panCancer.php

3 https://circinteractome.nia.nih.gov/

## References

[B1] Amininia S. Mohammad H. Mahboubeh E. Mohammad A. M. Seyed M. H. Mohsen T. (2014). Association between CCNE1 polymorphisms and the risk of breast cancer in a sample of southeast Iranian population. *Med. Oncol.* 31:189. 10.1007/s12032-014-0189-z 25159285

[B2] Bonnici V. Russo F. Bombieri N. Pulvirenti A. Giugno R. (2014). Comprehensive reconstruction and visualization of non-coding regulatory networks in human. *Front. Bioeng. Biotechnol.* 2:69. 10.3389/fbioe.2014.00069 25540777 PMC4261811

[B3] Bousema J. E. Heineman D. J. Dijkgraaf M. G. W. Annema J. T. van den Broek F. J. C. (2020). Adherence to the mediastinal staging guideline and unforeseen N2 disease in patients with resectable non-small cell lung cancer: nationwide results from the dutch lung cancer audit - surgery. *Lung. Cancer* 142 51–58. 10.1016/j.lungcan.2020.02.008 32088606

[B4] Chen X. Mao R. Su W. Yang X. Geng Q. Guo C. (2020). Circular RNA circHIPK3 modulates autophagy via MIR124-3p-STAT3-PRKAA/AMPKalpha signaling in STK11 mutant lung cancer. *Autophagy* 16 659–671. 10.1080/15548627.2019.1634945 31232177 PMC7138221

[B5] Chen Z. Chen X. Lu B. Gu Y. Chen Q. Lei T. (2020). Up-regulated LINC01234 promotes non-small-cell lung cancer cell metastasis by activating VAV3 and repressing BTG2 expression. *J. Hematol. Oncol.* 13:7. 10.1186/s13045-019-0842-2 PMC6972004 31959200

[B6] Di X. Jin X. Li R. Zhao M. Wang K. (2019). CircRNAs and lung cancer: biomarkers and master regulators. *Life Sci.* 220 177–185. 10.1016/j.lfs.2019.01.055 30711537

[B7] Etemadmoghadam D. George J. Cowin P. A. Cullinane C. Kansara M. Australian Ovarian Cancer Study Group (2010). Amplicon-dependent CCNE1 expression is critical for clonogenic survival after cisplatin treatment and is correlated with 20q11 gain in ovarian cancer. *PLoS One* 5:e15498. 10.1371/journal.pone.0015498 21103391 PMC2980490

[B8] Gu C. Huang Z. Dai C. Wang Y. Ren Y. She Y. (2018). Prognostic analysis of limited resection versus lobectomy in stage IA small cell lung cancer patients based on the surveillance, epidemiology, and end results registry database. *Front. Genet.* 9:568. 10.3389/fgene.2018.00568 30524472 PMC6262036

[B9] Gu C. Xin S. Zhenyu H. Jiafei C. Jun Y. Jianxin S. (2020). A comprehensive study of construction and analysis of competitive endogenous RNA networks in lung adenocarcinoma. *Biochim. Biophys. Acta Proteins Proteom.* 1868:140444. 10.1016/j.bbapap.2020.140444 32423886

[B10] Gu C. Xufeng P. Rui W. Yuan L. Xuxia S. Jianxin S. (2016). Analysis of mutational and clinicopathologic characteristics of lung adenocarcinoma with clear cell component. *Oncotarget* 7 24596–24603. 10.18632/oncotarget.8258 27013585 PMC5029726

[B11] Haghi M. Taha M. F. Javeri A. (2019). Suppressive effect of exogenous miR-16 and miR-34a on tumorigenesis of breast cancer cells. *J. Cell Biochem.* 120 13342–13353. 10.1002/jcb.28608 30916815

[B12] Han Z. Zhang Y. Yang Q. Liu B. Wu J. Zhang Y. (2015). miR-497 and miR-34a retard lung cancer growth by co-inhibiting cyclin E1 (CCNE1). *Oncotarget* 6 13149–13163. 10.18632/oncotarget.3693 25909221 PMC4537005

[B13] Kim S. H. Jin-Nyoung H. Hyunjin J. Sang C. L. Sang E. L. Sung-Kyu H. (2016). Upregulated expression of BCL2, MCM7, and CCNE1 indicate cisplatin-resistance in the set of two human bladder cancer cell lines: T24 cisplatin sensitive and T24R2 cisplatin resistant bladder cancer cell lines. *Investig. Clin. Urol.* 57 63–72. 10.4111/icu.2016.57.1.63 26966728 PMC4778756

[B14] Kong Z. Wan X. Lu Y. Zhang Y. Huang Y. Xu Y. (2019). Circular RNA circFOXO3 promotes prostate cancer progression through sponging miR-29a-3p. *J. Cell Mol. Med.* 24 799–813. 10.1111/jcmm.14791 31733095 PMC6933405

[B15] Kong Z. Wan X. Lu Y. Zhang Y. Huang Y. Xu Y. (2020). Circular RNA circFOXO3 promotes prostate cancer progression through sponging miR-29a-3p. *J. Cell Mol. Med.* 24 799–813. 10.1111/jcmm.14791 31733095 PMC6933405

[B16] Lang W. Wang J. Ma X. Zhang N. Li H. Cui P. (2019). Identification of shared genes between ischemic stroke and Parkinson’s Disease using genome-wide association studies. *Front. Neurol.* 10:297. 10.3389/fneur.2019.00297 30984102 PMC6447678

[B17] Lee J. Ku B. M. Shim J. H. La Choi Y. Sun J. M. Lee S. H. (2020). Characteristics and outcomes of RET-rearranged Korean non-small cell lung cancer patients in real-world practice. *Jpn. J. Clin. Oncol.* 50 594–601. 10.1093/jjco/hyaa019 32083304

[B18] Li Y. Gong P. Hou J. X. Huang W. Ma X. P. Wang Y. L. (2018). miR-34a regulates multidrug resistance via positively modulating OAZ2 signaling in colon Cancer cells. *J. Immunol. Res.* 2018:7498514. 10.1155/2018/7498514 PMC6098920 30175154

[B19] Liu C. Zhang Z. Qi D. (2019). Circular RNA hsa_circ_0023404 promotes proliferation, migration and invasion in non-small cell lung cancer by regulating miR-217/ZEB1 axis. *Onco Targets Ther.* 12 6181–6189. 10.2147/ott.s201834 31496723 PMC6689096

[B20] Liu H. Yin J. Wang H. Jiang G. Deng M. Zhang G. (2015). FOXO3a modulates WNT/beta-catenin signaling and suppresses epithelial-to-mesenchymal transition in prostate cancer cells. *Cell Signal.* 27 510–518. 10.1016/j.cellsig.2015.01.001 25578861

[B21] Lu H. Han X. Ren J. Ren K. Li Z. Sun Z. (2020). Circular RNA HIPK3 induces cell proliferation and inhibits apoptosis in non-small cell lung cancer through sponging miR-149. *Cancer Biol. Ther.* 21 113–121. 10.1080/15384047.2019.1669995 31597523 PMC7012091

[B22] Luo F. Zhao Y. Liu J. (2020). Cell adhesion molecule 4 suppresses cell growth and metastasis by inhibiting the Akt signaling pathway in non-small cell lung cancer. *Int. J. Biochem. Cell Biol.* 123:105750. 10.1016/j.biocel.2020.105750 32325280

[B23] Luo Y. Chen J. J. Lv Q. Qin J. Huang Y. Z. Yu M. H. (2019). Long non-coding RNA NEAT1 promotes colorectal cancer progression by competitively binding miR-34a with SIRT1 and enhancing the Wnt/beta-catenin signaling pathway. *Cancer Lett.* 440-441 11–22. 10.1016/j.canlet.2018.10.002 30312725

[B24] Marin I. Efrat O. Jair B. Nadia P. Marina P. Camila A. (2020). MiR-21, EGFR and PTEN in non-small cell lung cancer: an in situ hybridisation and immunohistochemistry study. *J. Clin. Pathol.* 10.1309/AJCPST1CTHZS3PSZ [Epub ahead of print]. 32060074

[B25] Neganova I. Vilella F. Atkinson S. P. Lloret M. Passos J. F. von Zglinicki T. (2011). An important role for CDK2 in G1 to S checkpoint activation and DNA damage response in human embryonic stem cells. *Stem Cells* 29 651–659. 10.1002/stem.620 21319273

[B26] No Authors (2020). Non-small cell lung cancer responds to osimertinib plus savolitinib. *Cancer Discov.* 10:487. 10.1158/2159-8290.CD-RW2020-025 32060054

[B27] Noske A. Brandt S. Valtcheva N. Wagner U. Zhong Q. Bellini E. (2017). Detection of CCNE1/URI (19q12) amplification by in situ hybridisation is common in high grade and type II endometrial cancer. *Oncotarget* 8 14794–14805. 10.18632/oncotarget.11605 27582547 PMC5362444

[B28] Pils D. Bachmayr-Heyda A. Auer K. Svoboda M. Auner V. Hager G. (2014). Cyclin E1 (CCNE1) as independent positive prognostic factor in advanced stage serous ovarian cancer patients - a study of the OVCAD consortium. *Eur. J. Cancer* 50 99–110. 10.1016/j.ejca.2013.09.011 24176298

[B29] Ruan L. Chen J. Ruan L. Tan A. Wang P. (2018). miR-34a inhibits tumorigenesis of NSCLC via targeting SIRT6. *Int. J. Clin. Exp. Pathol.* 11 1135–1145. 31938208 PMC6958161

[B30] Scheff R. J. Schneider B. J. (2013). Non-small-cell lung cancer: treatment of late stage disease: chemotherapeutics and new frontiers. *Semin. Intervent. Radiol.* 30 191–198. 10.1055/s-0033-1342961 24436536 PMC3710022

[B31] Shao Y. Zhong P. Sheng L. Zheng H. (2020). Circular RNA circDENND2A protects H9c2 cells from oxygen glucose deprivation-induced apoptosis through sponging microRNA-34a. *Cell Cycle* 19 246–255. 10.1080/15384101.2019.1708029 31878833 PMC6961690

[B32] Su C. Han Y. Zhang H. Li Y. Yi L. Wang X. (2018). CiRS-7 targeting miR-7 modulates the progression of non-small cell lung cancer in a manner dependent on NF-kappaB signalling. *J. Cell Mol. Med.* 2 3097–3107. 10.1111/jcmm.13587 29532994 PMC5980210

[B33] Su H. Defei Z. Yikun S. Yiwu D. (2019). Hypoxia-associated circDENND2A promotes glioma aggressiveness by sponging miR-625-5p. *Cell Mol. Biol. Lett.* 24:24. 10.1186/s11658-019-0149-x PMC6446274 30988674

[B34] Sun N. Zhang G. Liu Y. (2018). Long non-coding RNA XIST sponges miR-34a to promotes colon cancer progression via Wnt/beta-catenin signaling pathway. *Gene* 665 141–148. 10.1016/j.gene.2018.04.014 29679755

[B35] Wang F. Fu X. D. Zhou Y. Zhang Y. (2009). Down-regulation of the cyclin E1 oncogene expression by microRNA-16-1 induces cell cycle arrest in human cancer cells. *BMB Rep.* 42 725–730. 10.5483/bmbrep.2009.42.11.725 19944013 PMC6604625

[B36] Wang X. He C. Yang Z. Li S. Qiao L. Fang L. (2019). Dysregulation of long non-coding RNA SNHG12 alters the viability, apoptosis, and autophagy of prostate cancer cells by regulating miR-195/CCNE1 axis. *Int. J. Clin. Exp. Pathol.* 12 1272–1283. 31933941 PMC6947053

[B37] Warr M. R. innewies M. Flach J. Reynaud D. Garg T. Malhotra R. (2013). FOXO3A directs a protective autophagy program in haematopoietic stem cells. *Nature* 494 323–327. 10.1038/nature11895 23389440 PMC3579002

[B38] Wei S. Yuanyuan Z. Yanru J. Xiaojun L. Jian G. Yuanbing S. (2019). The circRNA circPTPRA suppresses epithelial-mesenchymal transitioning and metastasis of NSCLC cells by sponging miR-96-5p. *EBioMedicine* 44 182–193. 10.1016/j.ebiom.2019.05.032 31160270 PMC6604667

[B39] Yang R. Liu N. Chen L. Jiang Y. Shi Y. Mao C. (2019). GIAT4RA functions as a tumor suppressor in non-small cell lung cancer by counteracting Uchl3-mediated deubiquitination of LSH. *Oncogene* 38 7133–7145. 10.1038/s41388-019-0909-0 31417184

[B40] Yin D. Ogawa S. Kawamata N. Leiter A. Ham M. Li D. (2013). miR-34a functions as a tumor suppressor modulating EGFR in glioblastoma multiforme. *Oncogene* 32 1155–1163. 10.1038/onc.2012.132 22580610 PMC4085050

[B41] Yoshimura S. Gerondopoulos A. Linford A. Rigden D. J. Barr F. A. (2010). Family-wide characterization of the DENN domain Rab GDP-GTP exchange factors. *J. Cell Biol.* 191 367–381. 10.1083/jcb.201008051 20937701 PMC2958468

[B42] Yuan Z. Zhong L. Liu D. Yao J. Liu J. Zhong P. (2019). MiR-15b regulates cell differentiation and survival by targeting CCNE1 in APL cell lines. *Cell Signal.* 60 57–64. 10.1016/j.cellsig.2019.04.005 30965092

[B43] Zhang S. Liao K. Miao Z. Wang Q. Miao Y. Guo Z. (2019). CircFOXO3 promotes glioblastoma progression by acting as a competing endogenous RNA for NFAT5. *Neuro Oncol.* 21 1284–1296. 10.1093/neuonc/noz128 31504797 PMC6784278

[B44] Zhang Y. Yuan Y. Zhang Y. Cheng L. Zhou X. Chen K. (2020). SNHG7 accelerates cell migration and invasion through regulating miR-34a-Snail-EMT axis in gastric cancer. *Cell Cycle* 19 142–152. 10.1080/15384101.2019.1699753 31814518 PMC6927713

